# PDGF-CC underlies resistance to VEGF-A inhibition and combinatorial targeting of both suppresses pathological angiogenesis more efficiently

**DOI:** 10.18632/oncotarget.12843

**Published:** 2016-10-24

**Authors:** Lei Zheng, Chen Zhao, Yuxiang Du, Xianchai Lin, Yida Jiang, Chunsik Lee, Geng Tian, Jia Mi, Xianglin Li, Qishan Chen, Zhimin Ye, Lijuan Huang, Shasha Wang, Xiangrong Ren, Liying Xing, Wei Chen, Delong Huang, Zhiqin Gao, Shuping Zhang, Weisi Lu, Zhongshu Tang, Bin Wang, Rong Ju, Xuri Li

**Affiliations:** ^1^Center for Medical and Pharmaceutical Research, Binzhou Medical University, Yantai, Shandong, 264003, P. R. China; ^2^State Key Laboratory of Ophthalmology, Zhongshan Ophthalmic Center, Sun Yat-sen University, Guangzhou 510060, P. R. China; ^3^Department of Ophthalmology, the First Affiliated Hospital of Nanjing Medical University and State Key Laboratory of Reproductive Medicine, Nanjing Medical University, Nanjing 210029, P. R. China; ^4^Department of Cell Biology, Weifang Medical University, Weifang, 261053 P. R. China; ^5^Medical Imaging Institute, Shandong Province Characteristical Key Subject, Medical Imaging and Nuclear Medicine, Binzhou Medical University, Yantai, 264003 P. R. China

**Keywords:** angiogenesis, PDGF-CC, VEGF-A, drug resistance

## Abstract

Anti-VEGF-A therapy has proven to be effective for many neovascular diseases. However, drug resistance to anti-VEGF-A treatment can develop. Also, not all patients with neovascular diseases are responsive to anti-VEGF-A treatment. The mechanisms underlying these important issues remain unclear. In this study, using different model systems, we found that inhibition of VEGF-A directly upregulated PDGF-CC and its receptors in multiple cell types in pathological angiogenesis *in vitro* and *in vivo*. Importantly, we further revealed that combinatorial targeting of VEGF-A and PDGF-CC suppressed pathological angiogenesis more efficiently than monotherapy. Given the potent angiogenic activity of PDGF-CC, our findings suggest that the development of resistance to anti-VEGF-A treatment may be caused by the compensatory upregulation of PDGF-CC, and combined inhibition of VEGF-A and PDGF-CC may have therapeutic advantages in treating neovascular diseases.

## INTRODUCTION

Anti-VEGF-A therapy has proven to be effective for the treatment of many neovascular diseases, such as cancer [1]. However, despite the great success, outstanding questions exist. For example, drug resistance to anti-VEGF-A therapy can develop after some time of treatment [2-4]. Moreover, not all patients with cancer or other neovascular diseases are responsive to anti-VEGF-A treatment [5, 6]. Unfortunately, the molecular mechanisms underlying these urgent issues remain unclear.

PDGF-CC is a relatively new member of the PDGF family. It was identified almost twenty years after the discovery of PDGF-A and PDGF-B [7, 8]. PDGF-CC is abundantly expressed by different types of cells, such as tumor cells [9, 10], endothelial cells (ECs) [11, 12], vascular smooth muscle cells (VSMCs) [12, 13], pericytes, fibroblasts [12, 14], and macrophages [15]. PDGF-CC binds to PDGFR-α and PDGFR-β [12], which are also expressed by many different cell types, including tumor cells [16, 17], VSMCs, pericytes, and fibroblasts [18], all of which play important roles in angiogenesis. We and others have shown that PDGF-CC is a potent angiogenic factor [14, 19-23]. Noteworthy, the angiogenic pathways induced by PDGF-CC appear to be largely VEGF-A-independent [24]. For example, Crawford *et al* [14] reported that tumor-associated fibroblasts that are deficient of VEGF-A but with abundant PDGF-CC expression can promote tumor angiogenesis efficiently [14]. However, it remains thus far unknown whether PDGF-CC expression is affected by anti-VEGF-A treatment and whether PDGF-CC is related to the acquired drug resistance to anti-VEGF-A treatment.

In this study, we investigated the expression status of PDGF-CC and its receptors after inhibition of VEGF-A using both cultured cells and a mouse model. We found that inhibition of VEGF-A increased the expressions of PDGF-CC and its receptors. Importantly, we further revealed that combined inhibition of VEGF-A together with PDGF-CC inhibited pathological angiogenesis more efficiently than monotherapy. Given the potent angiogenic nature of PDGF-CC, our data suggest that the upregulation of PDGF-CC induced by inhibition of VEGF-A may be responsible for the development of acquired drug resistance to anti-VEGF-A therapy, and combinatorial targeting of VEGF-A and PDGF-CC may have therapeutic advantages over monotherapy in treating neovascular diseases.

## RESULTS

### PDGF-CC is upregulated in specific mouse strains in pathological angiogenesis during choroidal neovascularization (CNV)

**Figure 1:**
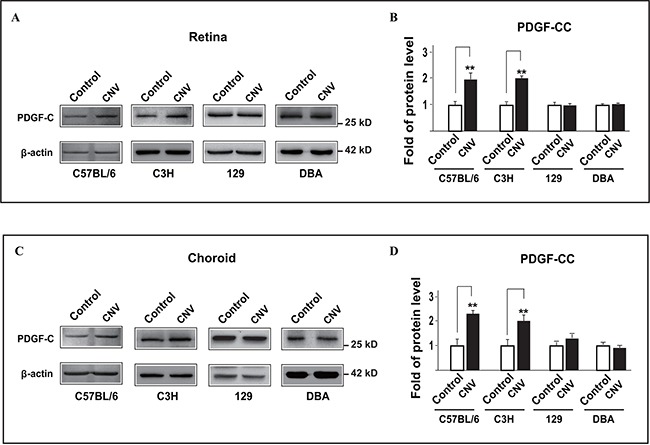
PDGF-CC is upregulated in pathological angiogenesis during choroidal neovascularization (CNV) in a strain-specific way in mice **A, B.** Western blots show that PDGF-CC protein levels were upregulated in the retinae three days after laser treatment in C57BL/6 and C3H but not 129 or DBA mice in laser-induced CNV. Quantifications of the bands in A are shown in B. **C, D.** Western blots show that PDGF-CC protein levels were upregulated in the choroids three days after laser treatment in C57BL/6 and C3H but not 129 or DBA mice in laser-induced CNV. Quantifications of the bands in C are shown in D. **P < 0.01.

We and others have shown that PDGF-CC is a potent angiogenic and survival factor [20, 22, 24-27]. However, it is unclear whether and how the expressions of PDGF-CC and its receptors are changed in pathological angiogenesis, such as in choroidal neovascularization (CNV) in different mouse strains. To address this, we utilized a laser-induced CNV mouse model and analyzed the protein levels of PDGF-CC using four mouse strains, including C57BL/6, C3H, 129 and DBA mice. We found that three days after laser treatment (the onset of CNV), PDGF-CC protein levels were upregulated in both the retinae (Figure 1A, 1B, n = 8, *P* < 0.01) and choroids (Figure 1C, 1D, n = 8, *P* < 0.01) in C57BL/6 and C3H mice. However, no significant change in PDGF-CC level was observed in 129 or DBA mice (Figure 1A-1D, n = 8, *P* > 0.05). To investigate the reason why the upregulation of PDGF-CC in the CNV model only took place in C57BL/6 but not in 129 mice, and since fibroblasts and macrophages are rich sources of PDGF-CC [14, 15], we investigated whether there could be a differential recruitment of these cells in different mouse strains. At day three after laser treatment, immunofluoresence staining using SMA and Iba1 as markers for fibroblasts and macrophages respectively, we found more Iba1 staining in the CNVs of C57BL/6 but not 129 mice (supplementary Figure 1A, *P* < 0.05, n = 8 eyes each group), while no significant difference was found in SMA staining (supplementary Figure 1B, n = 8 eyes each group), indicating that the upregulation of PDGF-CC in C57BL/6 mice was likely mainly due to the Iba1^+^ cells.

### PDGFR-α and PDGFR-β are upregulated at different time points during choroidal neovascularization

**Figure 2:**
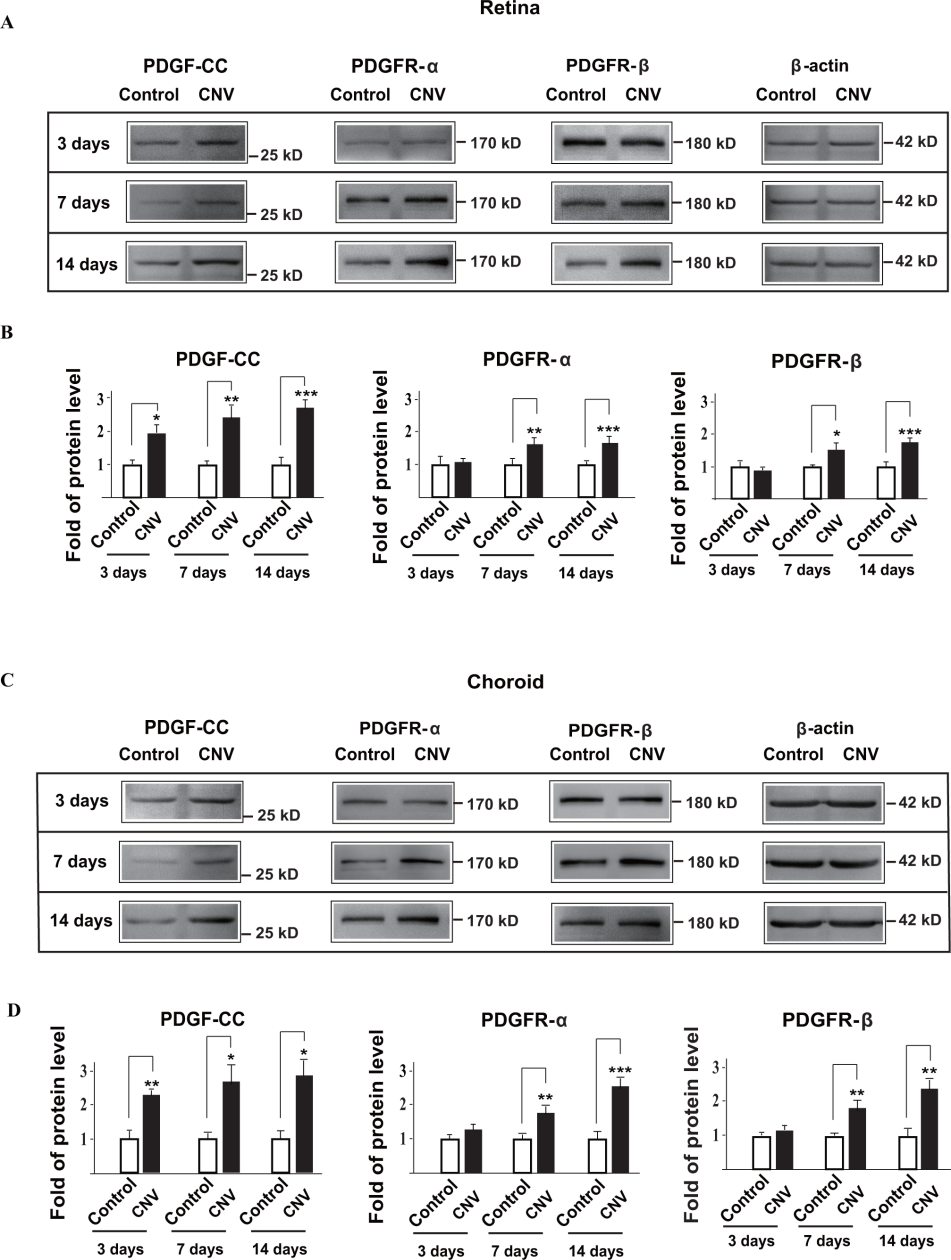
PDGFR-α and PDGFR-β are upregulated during choroidal neovascularization at different time points **A, B.** Western blots show that the expressions of PDGFR-α and PDGFR-β in the retinae were upregulated seven and fourteen days after laser-treatment in laser-induced mouse CNV. The expression of PDGF-CC was upregulated in the retinae earlier three days after laser-treatment. Quantifications of the bands in A are shown in B. **C, D.** Western blots show that the expressions of PDGFR-α and PDGFR-β in the choroids are upregulated seven and fourteen days after laser-treatment in laser-induced mouse CNV. The expression of PDGF-CC is upregulated earlier in the choroids at three days after laser-treatment. Quantifications of the bands in C are shown in D. *P < 0.05, **P < 0.01, ***P < 0.001.

PDGF-CC binds to PDGFR-α and PDGFR-β[19, 28]. However, it is unclear whether and when the expressions of PDGFR-α and PDGFR-β were changed in choroidal neovascularization. We therefore investigated these using C57BL/6 mice. Western blots showed that the expression levels of PDGFR-α and PDGFR-β were upregulated at seven and fourteen days after laser-induced CNV in both the retinae (Figure 2A, 2B, n=8, *P* < 0.05, 0.01 or 0.001) and choroids (Figure 2C, 2D, n=8, *P* < 0.05, 0.01 or 0.001), after the upregulation of PDGF-CC three days after laser treatment (Figure 2A-2D).

### VEGF-A neutralizing antibody upregulates PDGF-CC and its receptors in CNV

**Figure 3:**
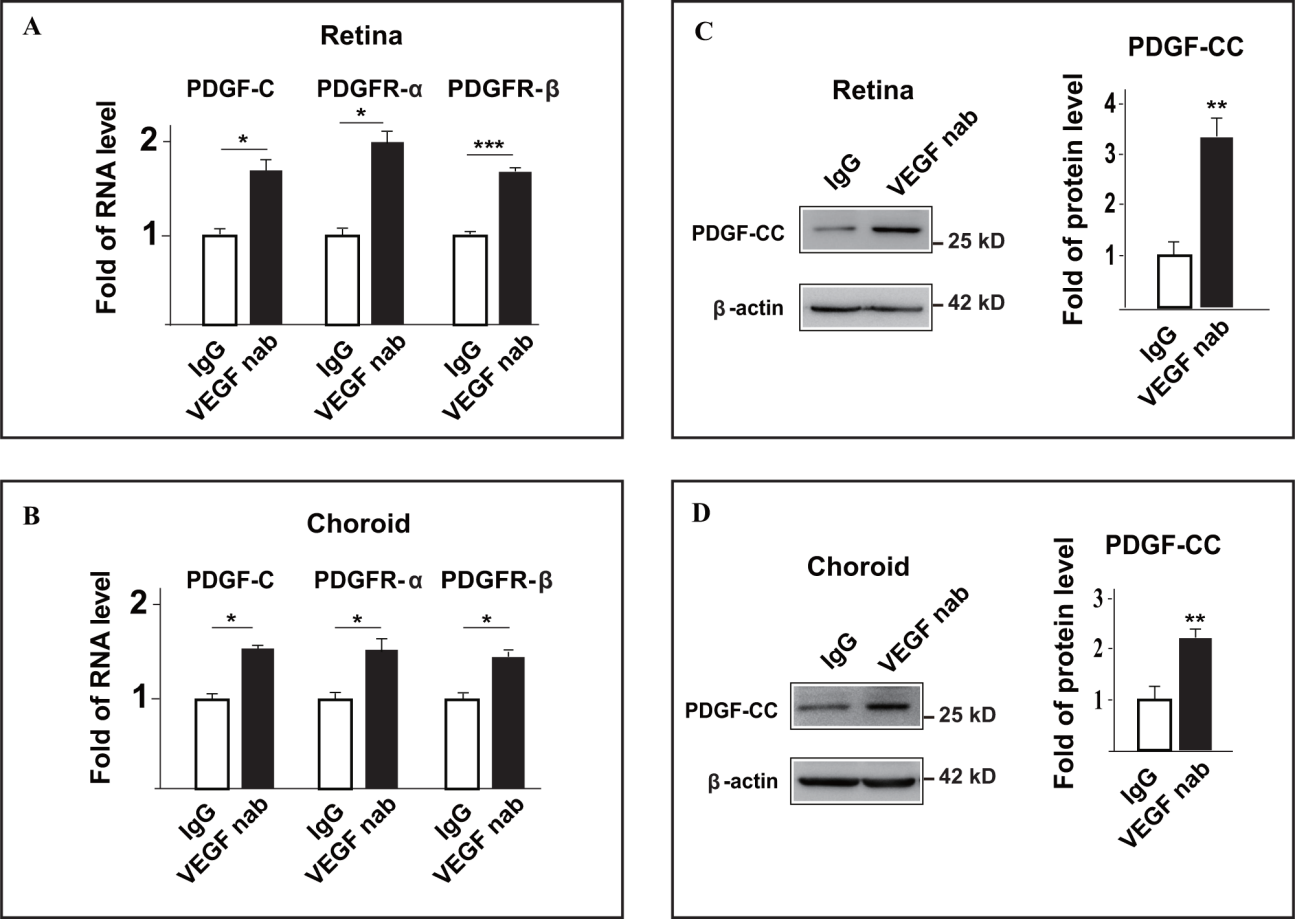
Inhibition of VEGF-A by neutralizing antibody upregulates PDGF-CC and its receptors *in vivo* in pathological angiogenesis **A.** Real-time PCR shows that intravitreal injection of VEGF-A neutralizing antibody (VEGF nab) increased the RNA levels of PDGF-C, PDGFR-α and PDGFR-β in the retinae four days after injection in laser-induced CNV. **B.** Real-time PCR shows that intravitreal injection of VEGF-A neutralizing antibody (VEGF nab) increased the RNA levels of PDGF-C, PDGFR-α and PDGFR-β in the choroids four days after injection in laser-induced CNV. **C.** Western blots show that intravitreal injection of VEGF-A neutralizing antibody (VEGF nab) increased the protein level of PDGF-CC in the retinae four days after injection in laser-induced CNV. **D.** Western blots show that intravitreal injection of VEGF-A neutralizing antibody (VEGF nab) increased the protein level of PDGF-CC in the choroids four days after injection in laser-induced CNV. *P < 0.05, **P < 0.01, ***P < 0.001.

Anti-VEGF-A therapy has been widely used to treat patients with cancer or other neovascular diseases. However, after a certain period of anti-VEGF-A treatment, many patients develop drug resistance. The mechanism for this acquired drug resistance remains unclear. To this end, we investigated whether inhibition of VEGF-A affected the expressions of PDGF-CC and its receptors in pathological angiogenesis using a mouse CNV model. Real-time PCR showed that intravitreal injection of VEGF-A neutralizing antibody (VEGF nab) increased the RNA levels of PDGF-C, PDGFR-α and PDGFR-β in the retinae four days after VEGF nab injection in C57BL/6 mice (Figure 3A, n=8, *P* < 0.05 or 0.001). This was also true for the choroids (Figure 3B, n=8, *P* < 0.05). Indeed, Western blot confirmed higher PDGF-CC protein levels in the retinae (Figure 3C, n=8, *P* < 0.01) and choroids (Figure 3D, n=8, *P* < 0.01) after intravitreal injection of VEGF-A nab. Thus, inhibition of VEGF-A by neutralizing antibody upregulated the expressions of PDGF-CC and its receptors in pathological angiogenesis in CNV.

### Multiple cell types are responsible for the upregulation of PDGF-CC after inhibition of VEGF-A

**Figure 4:**
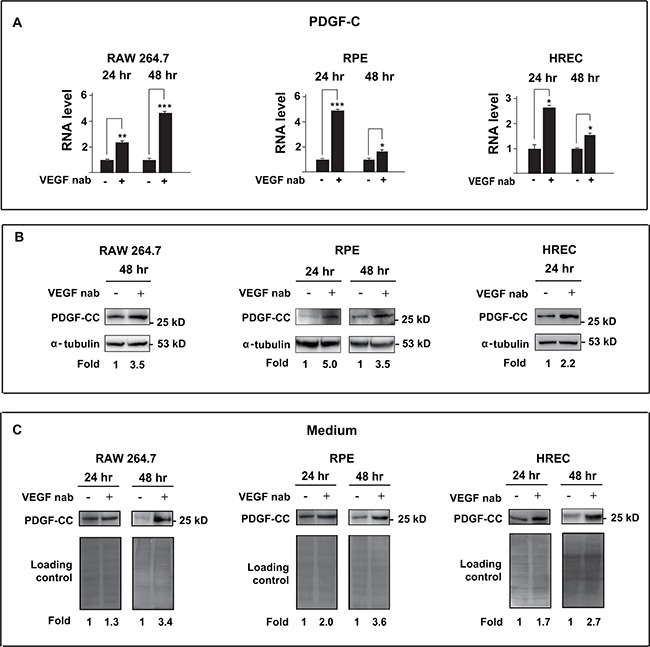
Inhibition of VEGF-A by neutralizing antibody upregulates PDGF-CC in multiple cell types **A.** Real-time PCR shows that inhibition of VEGF-A by neutralizing antibody (VEGF nab) increased the RNA level of PDGF-C in mouse macrophage RAW264.7 cells, human retinal pigment epithelial ARPE-19 cells and human retinal endothelial cells (HRECs) at different time points. **B.** Western blots confirmed higher PDGF-CC protein levels in the cells treated with VEGF-A neutralizing antibody (VEGF nab) at different time points. **C.** Greater amount of PDGF-CC protein was detected by Western blots in the conditioned serum-free medium from cells treated with VEGF nab. Ponceau S staining is used as loading controls for the conditioned media. *P < 0.05, **P < 0.01, ***P < 0.001.

We next investigated the cell types that are responsive for the upregulation of PDGF-CC after inhibition of VEGF-A using cultured endothelial cells [29], macrophages [30, 31] and retinal pigment epithelial cells [32], since they play important roles in pathological angiogenesis. Real-time PCR showed that inhibition of VEGF-A by neutralizing antibody (VEGF nab) increased the RNA level of PDGF-C in mouse macrophage RAW264.7 cells, human retinal pigment epithelial ARPE-19 cells and human retinal endothelial cells (HRECs) at different time points (Figure 4A, n=3, *P* < 0.05, 0.01 or 0.001). Indeed, Western blot confirmed the higher levels of PDGF-CC protein in the cell lysates (Figure 4B) and serum-free conditioned medium (Figure 4C), since PDGF-CC is a secreted protein [33]. Our results thus demonstrate that multiple cell types are responsible for the upregulation of PDGF-CC after inhibition of VEGF-A.

### Upregulation of PDGF-CC by inhibition of VEGF-A is VEGFR-2 and NP-1 independent

**Figure 5:**
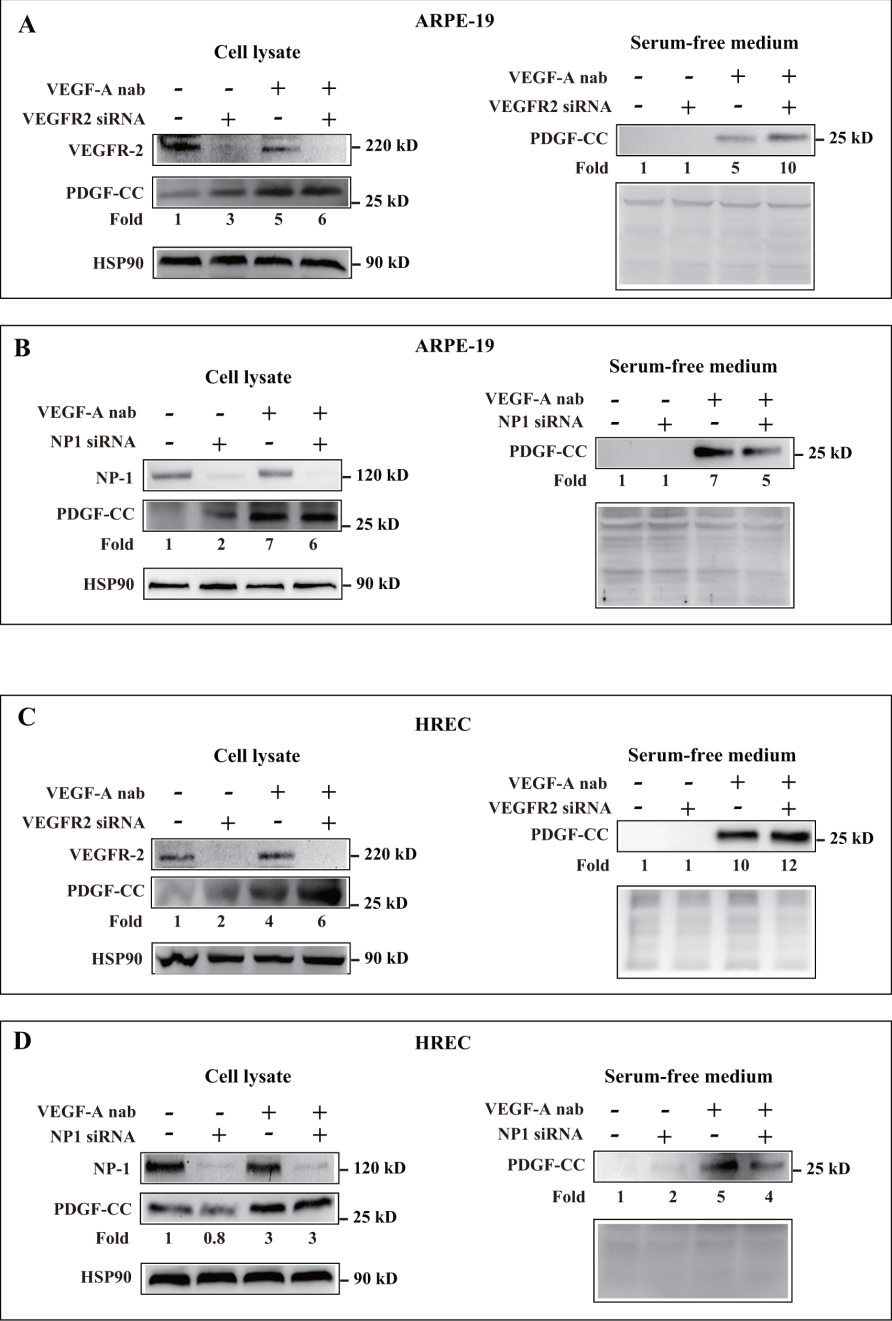
Upregulation of PDGF-CC by inhibition of VEGF-A is VEGFR-2 and NP-1 independent **A-D.** Western blots show that VEGFR-2 and NP1 are expressed in ARPE-19 and HREC cells and were successfully deleted by their respective siRNA. Western blots show that knockdown of VEGFR-2 and NP1 did not abrogate the upregulation of PDGF-CC by inhibition of VEGF-A with neutralizing antibody in cell lysates or serum-free medium. Ponceau S staining is used as loading controls for the conditioned media.

We next analyzed the expression levels of the receptors for VEGF-A, VEGFR-2 and NP1, in the cell lines and tested whether the knockdown of them affected PDGF-CC upregulation. Western blots showed that VEGFR-2 and NP1 were expressed in ARPE-19 and HREC cells and were successfully deleted by siRNA (Figure 5A-5D). We found that knockdown of VEGFR-2 and NP1 did not abrogate the upregulation of PDGF-CC by inhibition of VEGF-A with neutralizing antibody as shown by Western blots using both cell lysates and serum-free medium (Figure 5A-5D), suggesting that the upregulation of PDGF-CC by inhibition of VEGF-A is VEGFR-2 and NP-1 independent.

### VEGF-A knockdown by siRNA/shRNA upregulates PDGF-CC and PDGFRs *in vitro* and *in vivo*

**Figure 6:**
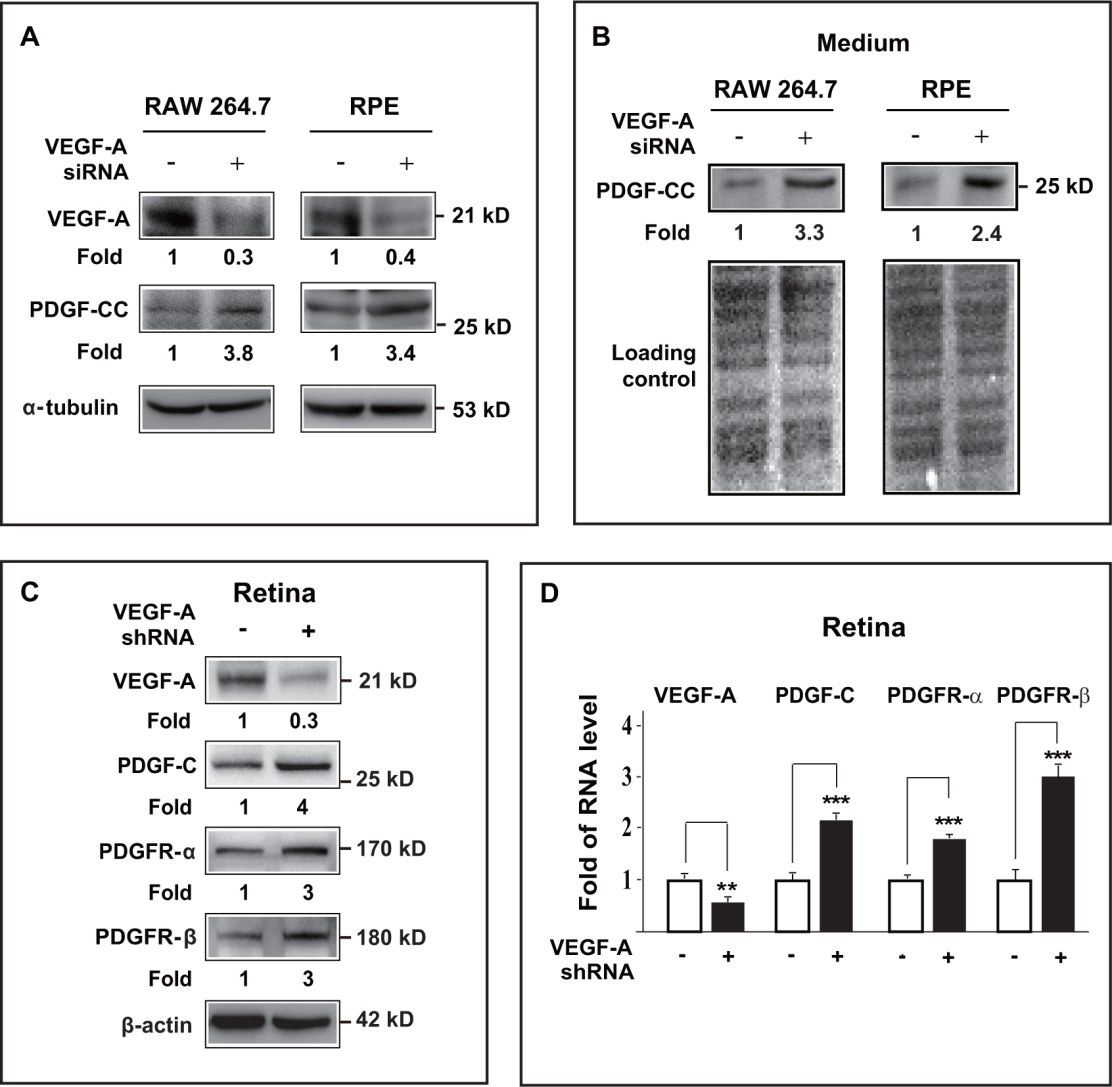
VEGF-A knockdown by siRNA/shRNA upregulated PDGF-CC and PDGFRs *in vitro* and *in vivo* **A.** Western blots show that in cultured Raw264.7 macrophage and ARPE-19 cells, VEGF-A knockdown by siRNA reduced VEGF-A expression to about 30-40 % of normal level (upper panel). Loss of VEGF-A subsequently resulted in higher PDGF-CC protein levels in these cells (middle panel). **B.** Western blots confirmed greater amount of secreted PDGF-CC protein (upper panel) in conditioned serum-free medium from Raw264.7 and ARPE-19 cells treated with VEGF-A siRNA. Ponceau S staining is used as loading controls for the conditioned media. **C.** Western blots show that in mouse retinae, VEGF-A knockdown by shRNA reduced VEGF-A expression level (upper panel) and increased the protein levels of PDGF-CC, PDGFR-α and PDGFR-β (lower panels). **D.** Real-time PCR reveals higher RNA levels of PDGF-C, PDGFR-α and PDGFR-β in mouse retinae after VEGF-A knockdown by shRNA. **P < 0.01, ***P < 0.001.

We subsequently engaged yet another approach to verify our findings by utilizing siRNA/shRNA to further investigate whether loss of VEGF-A could upregulate PDGF-CC*.* First, in cultured Raw264.7 macrophage and ARPE-19 cells, VEGF-A knockdown by siRNA reduced the expression of VEGF-A to about 30-40% of normal level (Figure 6A, upper panel). Loss a VEGF-A subsequently increased PDGF-CC protein levels in both the cell lysates (Figure 6A, middle panel) and in the conditioned serum-free medium (Figure 6B, upper panel).

Apart from cultured cells, *in vivo* assays using mouse retinae also showed VEGF-A knockdown by shRNA in mouse retina decreased the expression level of VEGF-A (Figure 6C, upper panel) with concomitant upregulation of PDGF-CC, PDGFR-α and PDGFR-β (Figure 6C). Consistently, real-time PCR also revealed increased RNA levels of PDGF-C, PDGFR-α and PDGFR-β in the retinae after VEGF-A knockdown (Figure 6D, n=6, *P* < 0.01 or 0.001). Thus, loss of VEGF-A upregulated the expressions of PDGF-CC, PDGFR-α and PDGFR-β both *in vitro* and *in vivo*.

### VEGF-A inhibition upregulates PDGF-CC in 129 mice with CNV

**Figure 7:**
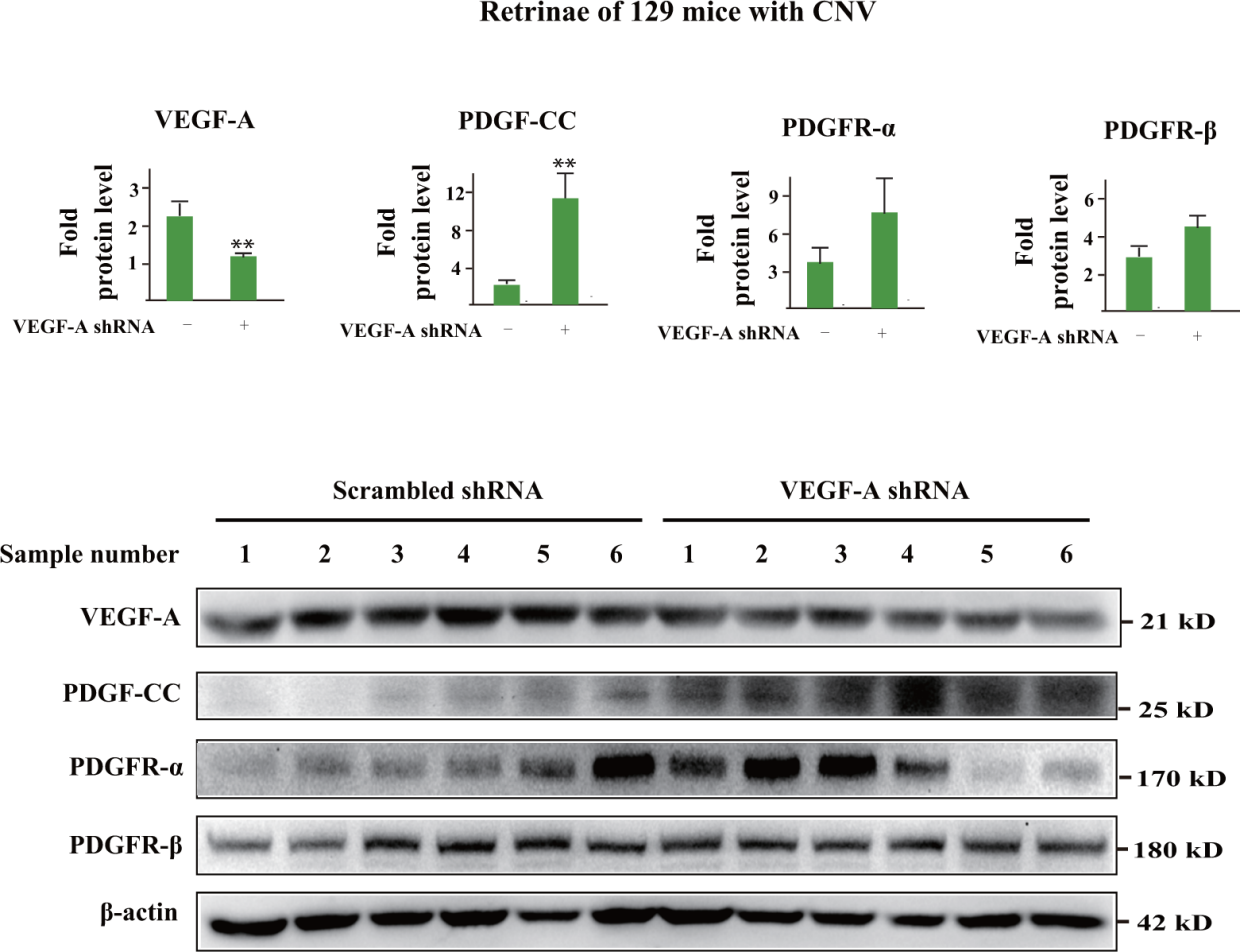
VEGF-A inhibition upregulates PDGF-CC in 129 mice with CNV Western blots show that in 129 mice, three days after laser induced-CNV and VEGF-A knockdown by shRNA, VEGF-A protein levels decreased in the retinae with a concomitant increase of PDGF-CC protein levels. Thus, even though there was no initial upregulation of PDGF-CC in the retinae of 129 mice in CNV, loss of VEGF-A also upregulated PDGF-CC.

We further investigated whether the upregulation of PDGF-CC after VEGF-A inhibition also took place in 129 mice, in which there was no initial increase of PDGF-CC in CNV. Western blots showed that three days after laser induced-CNV and VEGF-A knockdown by shRNA, VEGF-A protein levels decreased in the retinae of 129 mice with a concomitant increase of PDGF-CC protein levels (Figure 7, n=6, *P* < 0.01). Thus, even though there was no initial upregulation of PDGF-CC in 129 mice during CNV, loss of VEGF-A also upregulated PDGF-CC.

### Combinatorial targeting of VEGF-A and PDGF-CC suppresses pathological angiogenesis more efficiently

**Figure 8:**
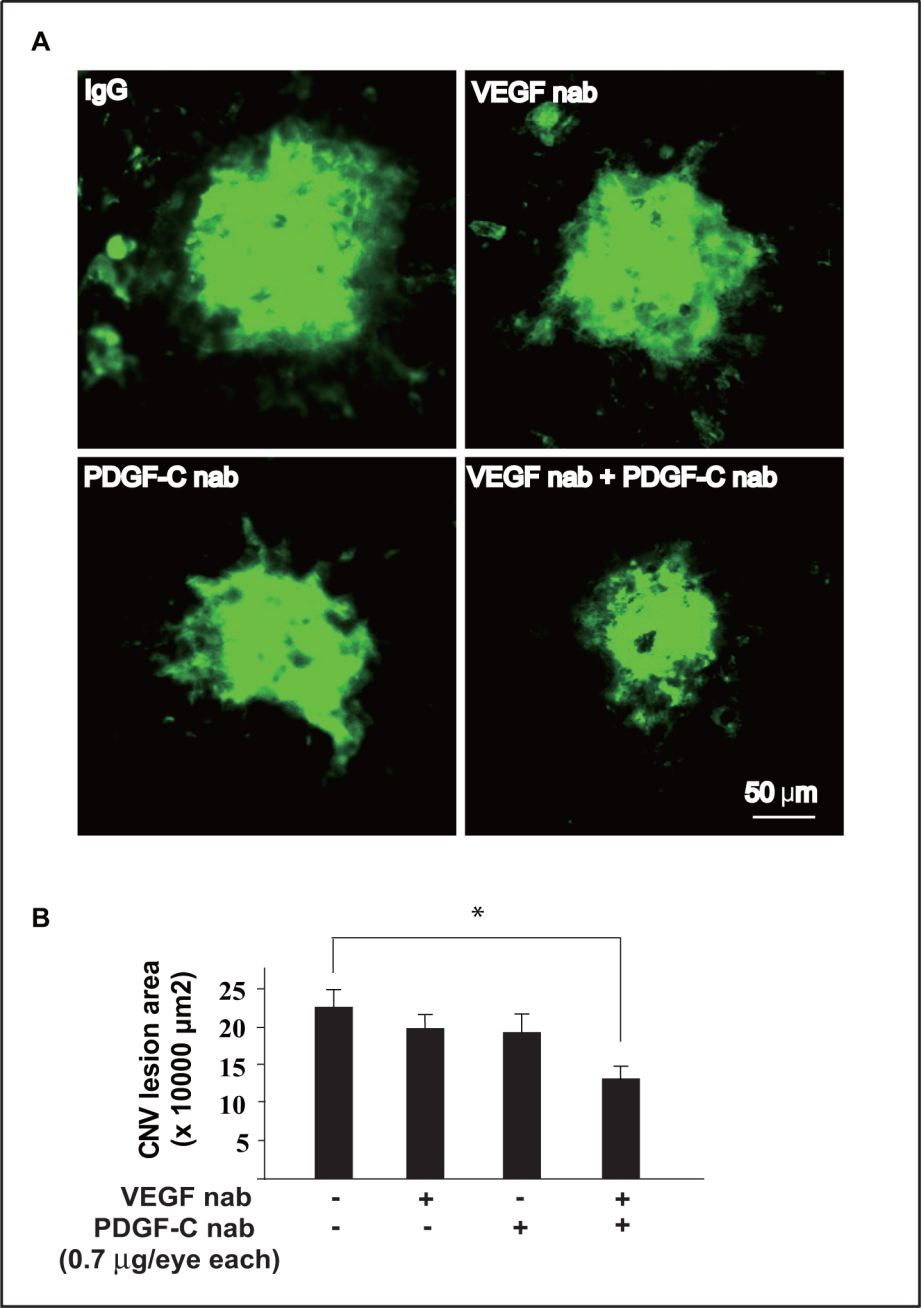
Combined targeting of VEGF-A and PDGF-CC inhibited pathological angiogenesis more efficiently **A, B.** When VEGF-A or PDGF-CC neutralizing antibody was administered alone respectively at a very low dose (0.7 μg/eye), none inhibit pathological angiogenesis in a laser-induced mouse CNV model. However, when the same low dose of PDGF-CC and VEGF-A neutralizing antibodies were administered together, pathological angiogenesis was significantly inhibited. Quantifications of the images in A are shown in B. *P < 0.05

Since inhibition of VEGF-A upregulated PDGF-CC expression, and PDGF-CC was a potent angiogenic factor [19, 20, 22], we hypothesized that blocking PDGF-CC and VEGF-A simultaneously might be able to suppress pathological angiogenesis more efficiently than monotherapy alone. We tested this hypothesis using a laser-induced mouse CNV model and found that when VEGF-A or PDGF-CC neutralizing antibody was administered alone at a very low dose (0.7 μg/eye), neither inhibited CNV (Figure 8A, 8B, n=8, *P* > 0.05). However, when the same low dose of PDGF-CC and VEGF-A neutralizing antibodies were administered together, CNV formation was significantly inhibited (Figure 8A, 8B, n=8, *P* < 0.05), demonstrating that combined inhibition of PDGF-CC and VEGF-A may have therapeutic advantages in inhibiting pathological angiogenesis.

## DISCUSSION

Anti-VEGF-A therapy has achieved great success in treating cancer and other neovascular diseases. However, outstanding questions exist. For example, drug resistance or tachyphylaxis can develop in many patients after a certain time of treatment [2-4, 34]. Also, some patients with neovascular diseases are not responsive to anti-VEGF-A therapy [5, 6]. Importantly, the molecular mechanisms underlying these important issues remain unclear. In this study, we investigated the expressions of PDGF-CC and its receptors after inhibition of VEGF-A using different model systems. We found that both *in vitro* and *in vivo*, inhibition of VEGF-A by different approaches upregulated the expressions of PDGF-CC and its receptors. Moreover, and importantly, combined inhibition of PDGF-CC and VEGF-A simultaneously suppressed pathological angiogenesis more efficiently than monotherapy. Given the potent angiogenic nature of PDGF-CC, its compensatory upregulation following inhibition of VEGF-A may be at least one of the mechanisms for the development of drug resistance to anti-VEGF-A therapy.

PDGF-CC is abundantly expressed by different types of cells, including tumor cells [9, 10], vascular ECs [11-13], vascular SMCs [12, 13, 35-37], pericytes, fibroblasts [12, 14] and macrophages [15], all of which play important roles in pathological angiogenesis. VEGF-A blockages have been routinely used in the clinic to treat cancer and other neovascular diseases. However, it remains unknown whether inhibition of VEGF-A affects PDGF-CC expression. In this study, we reveal for the first time that inhibition of VEGF-A upregulates PDGF-CC and its receptors in multiple cell types *in vitro* and in pathological angiogenesis *in vivo*. Our finding demonstrates that pathological angiogenesis involves complex pathways, and inhibition of VEGF-A can activate compensatory or alternative angiogenic pathways. Thus, for better anti-angionenic effect, it may be critical to inhibit such compensatory pathways together with VEGF-A.

Independent groups have shown that PDGF-CC is a potent angiogenic factor [14, 19, 20, 22, 38]. Noteworthy, PDGF-CC-induced angiogenesis appears to be largely VEGF-A-independent. For example, under conditions of VEGF-A deficiency, fibroblasts expressing PDGF-CC still induced robust tumor angiogenesis [14]. Indeed, unlike VEGF-A, which targets mainly endothelial cells, PDGF-CC regulates the functions of a broad spectrum of cell types, such as tumor cells, vascular smooth muscle cells, macrophages, RPE cells, fibroblasts [22], and endothelial cells [20]. Indeed, consistently, we found in this study that combined inhibition of PDGF-CC together with VEGF-A renders a better outcome in suppressing pathological angiogenesis than inhibition of VEGF-A or PDGF-CC alone respectively.

Interestingly, we found in this study that the upregulation of PDGF-CC during pathological angiogenesis is strain-specific in mice. Among four different mouse strains (C57BL/6, C3H, 129 and DBA), the upregulation of PDGF-CC in CNV was only observed in C57BL/6 and C3H mice, but not in 129 or DBA strains. This demonstrates that genetic background plays an important role in regulating the expression of PDGF-CC. Even though further studies are needed to test whether this is also true in humans, it may provide an explanation why a subpopulation of patients with pathological angiogenesis are not responsive to anti-VEGF-A therapy. Noteworthy, despite of the lack of initial upregulation of PDGF-CC in CNV, inhibition of VEGF-A also increased PDGF-CC expression in these mice, suggestion that the inhibition-of-VEGF-A-induced upregulation of PDGF-CC may be a general event in pathological angiogenesis.

In summary, in this study, we reveal that inhibition of VEGF-A directly upregulates the expressions of PDGF-CC and its receptors in pathological angiogenesis. Moreover, we report that blocking VEGF-A and PDGF-CC simultaneously is more efficient in suppressing pathological angiogenesis than targeting only one of them respectively. Our finding advocate combined inhibition of VEGF-A and PDGF-CC as a therapeutic strategy to treat patients with neovascular diseases.

## MATERIALS AND METHODS

### Mice

6-8 weeks old female mice of different strains (C57BL/6, C3H, 129 and DBA) were purchased from Vital River, Beijing, China. All animal experiments were approved by the Animal Care and Use Committee at the Zhongshan Ophthalmic Center and were performed according to the regulations of Sun Yat-sen University in Guangzhou, China.

### Laser-induced mouse CNV model

The laser-induced mouse CNV model was performed as described previously [22, 39, 40]. Briefly, four laser spots were made (75μm spot size, 75 ms, 90 mW power; OcuLight Infrared Laser System 810 nm, Iridex) in the area surrounding the optic disk in the eye. The eyes were harvested at 3, 7 and 14 days after laser-induced CNV for gene expression analysis.

For shRNA delivery into mouse eyes, the *in vivo*-jetPEI (Polyplus Transfection) transfection reagent was mixed with VEGF-A or scrambled shRNA according to the manufacturer's instructions [22]. Intravitreal injection of mouse VEGF-A shRNA (TRCN0000066822, 1 μg/eye, Open Biosystems) was performed together with the in vivo-jetPEI reagent. Age- and gender-matched control mice received intravitreal injection of scrambled shRNA (RHS6848, 1 μg/eye, Open Biosystems). The eyes were harvested for analysis at 3, 4 or 7 days after injection.

For simultaneous inhibition of VEGF-A and PDGF-CC, neutralizing antibody against mouse VEGF-A (0.7μg/eye, AF-493-NA, R&D Systems), neutralizing antibody against mouse PDGF-CC (0.7μg/eye, AF 1447, R&D Systems), or the combination of them at the same doses were injected intravitreally into mouse eyes. The first intravitreal injection was performed one day before laser treatment, and the second one three days after laser treatment.

For the measurement of CNV lesion areas, 7 days after laser treatment, the mice were euthanized and eyes harvested for immunostaining and imaging of the whole-mount choroids. The eyes were fixed in 4% paraformaldehyde for 2 hours. The cornea, crystalline lens and vitreous were removed and the retinae separated. The remaining eyecups were rinsed in PBS and incubated in isolectin GS-IB4 conjugated with Alexa Fluor 488 (1:500, Life Technologies, I21411) with 1% BSA overnight at 4°C. The eye cups were then washed with ice-cold PBS and flat-mounted with the RPE side facing up. Images were taken using a fluorescent microscope (Zeiss, German) and CNV areas analyzed using an image-analysis software (Zeiss, German).

### Iba1 and SMA immunofluorescence staining

Three days after laser treatment, the eyes were harvested and embedded into OCT for frozen sections at 10 μm thickness. All the sections were collected and analyzed. The sections were dried at room temperature for 10 min and then fixed in 4% paraformaldehyde for 20 min. After washing with PBS, the sections were permeabilized with 0.3% Tritonx-100 in PBS for 15 minutes and washed with PBS for three times. The sections were blocked with 5% BSA at room temperature for 1 hour. Then primary Iba1 (019-19741, WAKO) or SMA (A2547, Sigma) antibodies were diluted in 1% BSA and incubated with the sections overnight at 4°C. The sections were washed with PBS for 3 times followed by incubation with the respective secondary antibodies for 1 hour at room temperature protected from light. The sections were washed 3 times with PBS. DAPI was used to stain the nuclei. The sections were photographed and analyzed using a fluorescent microscope (Zeiss, German) and an image analysis software (Intel Integrated Performance Primitives, Intel IPP 6.0).

### Cell culture

The cells used in this study were obtained directly from cell banks and passaged in our laboratory for fewer than 6 months. Human retinal endothelial cells (HRECs) were purchased from Angio-Proteomie (Boston, MA, USA) and were characterized by the cell bank using Factor VIII staining, PECAM1 staining and uptake of Di-I-Ac-LDL. HRECs were cultured in endothelial cell media (ECM, ScienCell) supplemented with 5% fetal bovine serum (FBS), 1% penicillin/streptromycin with endothelial cell growth supplements (ECGS, ScienCell).

Human retinal pigment epithelial (RPE) cell line ARPE-19 was purchased from (ATCC, Manassas, VA, USA) and were authenticated by PCR using an STR Multi-amplification Kit (PowerPlexTM 16 HS System). The PCR products were analysed using a 3100 DNA Analyzer (Applied Biosystems®). Results showed that no loci has tri-alleles or tetra-alleles. Contamination of other human cell lines was not found. 100% matched cell lines were found in DSMZ and ATCC data banks, and the cell line is named as ARPE-19. The sample analyzed was shown to be a human cell line, and no contamination of cells from other species (*Cricetulus griseus, Macaca mulatta, Cercopithecus aethiops, Rattus norvegicus, Mus musculus, Bos Taurus, IC*) was found. The ARPE-19 cells were cultured in DMEM (Corning) supplemented 10% FBS and 1% penicillin/streptomycin.

The mouse macrophage cell line RAW264.7 was purchased (ATCC, Manassas, VA, USA) and cultured in RPMI-1640 (Corning) containing 10% FBS and 1% penicillin/streptomycin. The RAW264.7 cells were characterized by fluorescence staining using F4/80 antibody (AbD Serotec, MCA497) and CD11b antibody (BD Biosciences, 553308) as markers for macrophages. Briefly, the cells were washed with cold PBS and fixed with cold fixative (4% PFA) for 20 minutes. The cells were washed with cold PBS and permeabilized with 0.25% Triton in PBS for 5 minutes and washed with cold PBS for three times. The cells were blocked with 5% BSA in PBS for 30 minutes. Primary F4/80 or CD11b antibodies were diluted in 5% BSA and incubated with the cells for 2 hours at room temperature. The cells were washed with PBST for 3 times followed by incubation with secondary antibodies for 1 hour at room temperature. The cells were washed 3 times with PBST followed by DAPI staining and imaged using a microscope. More than 95% of the cells were F4/80^+^ and CD11b^+^ (data not shown).

All the cells were maintained at 37°C with 5% CO_2_. For VEGF-A neutralization, human or mouse cells were plated into 6-cm culture plates. At about 60% confluence, the cells were starved overnight and treated with VEGF-A neutralizing antibodies (AF-493-NA for mouse VEGF-A, AF-293-NA for human VEGF-A, 5 μg/ml; R&D Systems) or control IgG (AB-108-C, 5μg/ml; R&D Systems) for 24 and 48 hours with no or 1% FBS. The cells were harvested for analysis using RIPA buffer (50 mM Tris–HCl, 150 mMNaCl, 1% NP-40, 0.5% Sodium deoxycholate, and 0.1% SDS) with protease and phosphatase inhibitor cocktails (Thermo Scientific). TRIzol (Life Technology) was used for protein or RNA extraction. To detect secreted PDGF-CC protein, the conditioned serum-free medium from cultured cells were collected and subjected to precipitation using ice-cold trichloroacetic acid (TCA) as described [33]. Precipitated protein was washed several times with ice-cold acetone to remove residual TCA. SDS-PAGE assays were performed and followed by Western blot.

### siRNA transfection

The cells were plated into 6-well tissue culture plates and maintained until about 70-80% confluent. VEGF-A siRNA or control siRNAs were transfected into the cells using Lipofectamine RNAiMAX (Invitrogen) according to the manufacturer's protocols. After 24 hours, the transfection medium was replaced with serum-free medium and the cells were continuously incubated at 37°C with 5% CO_2_ for 24 hours and the cells were harvested for protein analysis.

### RNA isolation and real-time PCR

Total RNA was isolated using TRIzol reagent (Life Technology) according to the manufacturer's instruction. The quality of RNA was verified using 2% agarose gel electrophoresis (Seakem GTG; FMC Bioproducts, Rockland, ME) and spectrophotometry. One microgram of total RNA was reverse-transcribed into cDNA using a PrimeScript RT reagent Kit (TAKARA Biotechnology) according to the manufacturer's instruction. Real-time PCR was performed using qPCR SYBR® Green Master Mix (Roche) and a Roche 480 Light Cycler. The results were analyzed using a 480 Light Cycler software. All experiments were performed in triplicate and repeated twice. The PCR cycles consisted of an initial denaturation step at 95°C for 2 min, followed by 50 cycles of 95°C for 15 sec and 60°C for 60 sec. The primers used are listed in Table 1.

**Table 1:** Primers used for real-time PCR

Gene name	Orientation	Primer sequence (5′ to 3′)	Species
PDGF-C	forward	GAATTGGATTTCCGCATCT	mouse
PDGF-C	reserve	TAACCGCTTCACTTCCATTG	mouse
PDGF-C	forward	GAATCCAACCTGAGTAGTAA	human
PDGF-C	reserve	GACAATGTTGTAGTGGATGC	human
PDGFR-α	forward	CAAACCCTGAGACCACAATG	mouse
PDGFR-α	reserve	TCCCCCAACAGTAACCCAAG	mouse
PDGFR-β	forward	ACTACATCTCCAAAGGCAGCACCT	mouse
PDGFR-β	reserve	TGTAGAACTGGTCGTTCATGGGCA	mouse
β-actin	forward	GGAGCAATGATCTTGATCTTC	mouse
β-actin	reserve	GCCAACACAGTGCTGTCTGG	mouse
β-actin	forward	GGACTTCGAGCAAGAGATGG	human
β-actin	reserve	AGCACTGTGTTGGCGTACAG	human

### Western blot

Cultured cells were rinsed twice with ice-cold PBS and lysed in RIPA buffer (50 mM Tris-HCl, 150 mM NaCl, 1% NP-40, 0.5% Sodium deoxycholate and 0.1% SDS) with protease inhibitor and phosphatase inhibitor cocktails (Thermo Scientific). The cell lysates were centrifuged and the supernatants collected. Protein concentrations were determined using a DC™ protein assay kit (Bio-Rad, 5000111). Conditioned serum-free medium was collected and protein precipitated using ice-cold trichloroacetic acid (TCA) as described [41]. For retinae and choroids, the tissues were put into RIPA buffer with protease inhibitor and phosphatase inhibitor cocktails (Thermo Scientific) and homogenized, centrifuged, and protein samples collected. The protein samples with DTT (0.1M) were separated on a 10 % SDS-PAGE and transferred to a PVDF membrane. The membrane was incubated with the following primary antibodies at 4°C overnight: anti-VEGF-A (sc-53462, Santa Cruz), anti-PDGFR-α (sc-338, Santa Cruz), anti-PDGFR-β (sc-432, Santa Cruz), anti-PDGF-C (AF1560; AF1447, R&D), anti-NP1 (sc-5541, Santa Cruz), and anti-VEGFR2 (9698S, CST). The membranes were then incubated with horseradish peroxidase-conjugated secondary antibodies for one hour at room temperature and developed using Super-Signal Pico (Thermo Scientific) or Immobilon Western Chemiluminescent HRP substrate (Merck Millipore). The membranes were stripped and re-probed with a monoclonal antibody to β-actin or α-tubulin (Biovision) for loading control. The bands were visualized using a G: BOX (Syngene).

### Statistics

Two-tailed Student's t-test was used for statistical analysis using an Excel program. Difference was considered statistically significant when *P* < 0.05. Data are represented as mean ± s.e.m. of the number of the determinations.

## SUPPLEMENTARY MATERIALS FIGURE


