# Supplementary material for: PDGF-CC underlies resistance to VEGF-A inhibition and combinatorial targeting of both suppresses pathological angiogenesis more efficiently
Source: Oncotarget. 2016 Oct 24;7(47):77902–15. doi: 10.18632/oncotarget.12843 (PMC5363630; doi:10.18632/oncotarget.12843)
Supplement: Supplementary file 1 [file oncotarget-07-77902-s001.pdf]

PDGF-CC underlies resistance to VEGF-A inhibition and combinatorial targeting of both suppresses pathological angiogenesis more efficiently

SUPPLEMENTARY FIGURE

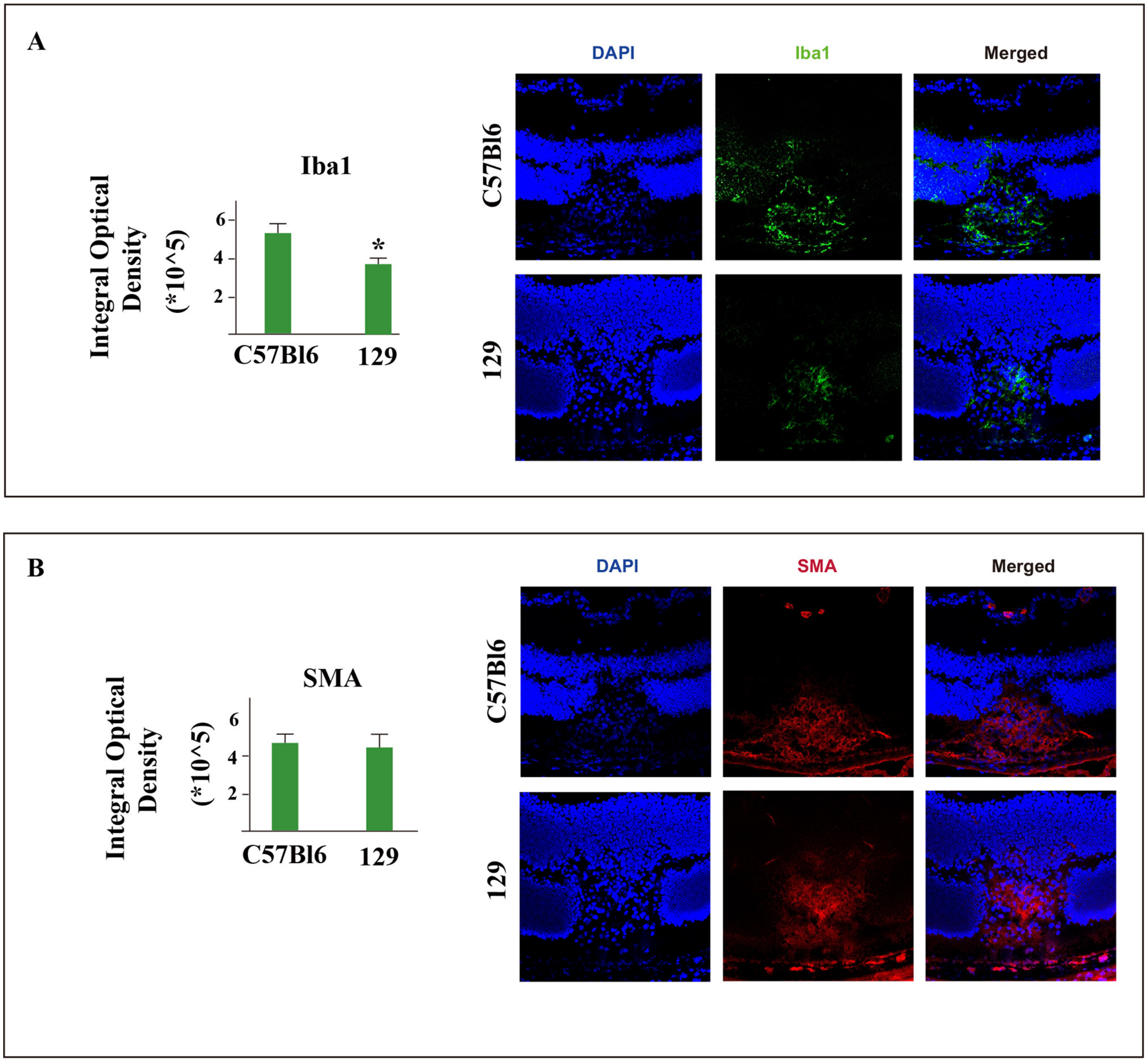

Supplementary Figure 1: More Iba1⁺ cells in the CNV lesions of C57Bl6 mice than those of 129 mice. A. At day three after laser treatment, immunofluoresence staining using Iba1 as a marker for macrophage shows more Iba1 staining in the CNV lesions of C57Bl6 mice than those of 129 mice. **B.** At day three after laser treatment, immunofluoresence staining using SMA as a marker for fibroblasts shows no significant difference in SMA staining in the CNV lesions of C57Bl6 and 129 mice.
